# Ferumoxytol magnetic resonance angiography: a dose-finding study in patients with chronic kidney disease

**DOI:** 10.1007/s00330-019-06137-4

**Published:** 2019-03-27

**Authors:** Sokratis Stoumpos, Martin Hennessy, Alex T. Vesey, Aleksandra Radjenovic, Ram Kasthuri, David B. Kingsmore, Patrick B. Mark, Giles Roditi

**Affiliations:** 1 Renal and Transplant Unit, Queen Elizabeth University Hospital, Glasgow, UK; 2 Institute of Cardiovascular and Medical Sciences, BHF Glasgow Cardiovascular Research Centre, University of Glasgow, Glasgow, G12 8TA UK; 3 Department of Radiology, Queen Elizabeth University Hospital, Glasgow, UK

**Keywords:** Ferumoxytol, Iron oxide, Magnetic resonance, MR angiography, Chronic kidney disease

## Abstract

**Objectives:**

Ferumoxytol is an alternative to gadolinium-based compounds as a vascular contrast agent for magnetic resonance angiography (MRA), particularly for patients with chronic kidney disease (CKD). However, dose-related efficacy data are lacking. We aimed to determine the optimal (minimum effective) dose of ferumoxytol for MRA in patients with CKD.

**Methods:**

Ferumoxytol-enhanced MRA (FeMRA) was performed at 3.0 T in patients with CKD after dose increments up to a total of 4 mg/kg. Image quality was assessed by contrast-to-noise ratio (CNR) and signal-to-noise ratio (SNR) in the abdominal aorta and inferior vena cava. Quadratic regression analyses were performed to estimate the effects of dose increments on CNR and SNR.

**Results:**

Twenty-three patients underwent FeMRA (mean age 60 [SD 13] years, 87% men, 48% had diabetic nephropathy) with cumulative doses of 0, 1, 2, 3 and 4 mg/kg of ferumoxytol. On regression analyses, a parabolic relationship was observed between ferumoxytol dose and signal with progressive signal loss using doses exceeding 4 mg/kg. A dose of 3 mg/kg achieved ≥ 75% of predicted peak CNR and SNR and images were deemed of excellent diagnostic quality.

**Conclusions:**

In patients with CKD undergoing FeMRA, a dose of 3 mg/kg provides excellent arterial and venous enhancement. The benefits of increasing the dose to a theoretically optimal value of 4 mg/kg appear to be negligible and likely of minimal, if any, diagnostic value.

**Key Points:**

*• Ferumoxytol is used off-label as an MRI contrast agent but dose-related data are lacking.*

*• In patients with CKD requiring MR angiography, a dose of 3 mg/kg provides excellent vascular enhancement.*

**Electronic supplementary material:**

The online version of this article (10.1007/s00330-019-06137-4) contains supplementary material, which is available to authorized users.

## Introduction

Ferumoxytol, an iron oxide nanoparticle compound with superparamagnetic properties, was originally designed as an intravascular contrast agent for magnetic resonance imaging (MRI) [1], although ultimately it was commercialised as an intravenous iron therapy for anaemia. Meanwhile, ferumoxytol is now increasingly used off-label by clinicians and researchers as an MRI contrast agent. Vascular imaging in patients with chronic kidney disease (CKD) can be challenging due to increased susceptibility to adverse events such as contrast-induced nephropathy (CIN) with iodine-based contrast agents and concerns regarding nephrogenic systemic fibrosis (NSF) with gadolinium-based contrast agents (GBCAs).

Ferumoxytol has high relaxivity at 1.5 T and 3.0 T [2] with theoretical advantages as a contrast agent for MRA. It is not filtered by the glomerulus but is removed from the circulation via macrophage phagocytosis with the remaining iron oxide particles taken up by the reticuloendothelial system of the liver, spleen and bone marrow being incorporated into body iron stores for red blood cell synthesis. Due to its large molecular weight of approximately 750 kD [3], ferumoxytol resides in the blood pool and does not diffuse out into the extracellular fluid space. Given its long intravascular half-life, it can potentially be used without the need for bolus timing with imaging in a ‘steady state’ when the arterial and venous vasculatures are equally enhanced.

In June 2009, the US Food and Drug Administration (FDA) approved ferumoxytol for parenteral treatment of iron deficiency anaemia (IDA) in adults with CKD and in February 2018 expanded the approval to include all eligible adults with IDA who cannot tolerate or have not responded to oral iron. Recently, ferumoxytol has gained appeal in vascular imaging and there are reports in the literature for its safe use and utility in both adult and paediatric patients with CKD [4–11]. However, dose-related efficacy studies are lacking with different doses having been reported in the literature, ranging from 120 mg of elemental iron as a bolus for angiographic assessment of arteriovenous fistulas [4] to 6 mg/kg for T2* renal perfusion mapping [12].

The aim of this study was to establish the optimal dose of ferumoxytol required for vascular imaging in patients with CKD.

## Materials and methods

### Study population

Patients older than 18 years of age with estimated glomerular filtration rate (eGFR) of < 30 mL/min/1.73 m^2^ (including patients on haemodialysis) requiring vascular imaging were included in this prospective, single-centre study. Patients were referred for ferumoxytol-enhanced MR angiography (FeMRA) if the clinician felt that an angiogram was required for one the following indications: characterisation of the aortoiliac vasculature prior to wait-listing for kidney transplantation, investigation of renal artery stenosis or to assess peripheral vascular disease (PVD). In all patients, standard contrast-enhanced imaging techniques were avoided due to potential risks of CIN or NSF. Dialysis patients were referred for FeMRA if they had residual renal function with a risk of decline or loss of function after use of iodine-based contrast agents or if there was prior evidence of extensive vascular calcifications which would impair CT images. Patients with standard contraindications to MRI (such as non-MRI-compatible pacemakers, severe claustrophobia and metal in the eyes), history of allergic reaction to any intravenous iron product, any conditions associated with iron overload and patients with active immune or inflammatory conditions (e.g. systemic lupus, rheumatoid arthritis) were excluded.

As ferumoxytol was increasingly used off-label in our centre as an alternative MRI contrast agent [11], this dose-finding study was performed to optimise our protocol. This was approved by the Clinical Governance Committee of the Diagnostics Directorate. The regional Research Ethics Committee ethics officer was consulted and confirmed that formal ethics committee approval was not required. Nevertheless, as this was an off-label use of the agent, informed consent was obtained from all subjects. Investigations were performed between 1 December 2015 and 30 June 2016.

### Baseline data

Age, gender and aetiology of established renal failure (ERF) were recorded. We retrieved data on last haemoglobin and serum creatinine prior to FeMRA and calculated the eGFR using the Chronic Kidney Disease Epidemiology Collaboration (CKD-EPI) equation [13]. All patients were weighed on the day of the scan to calculate the doses of ferumoxytol given.

### MRI protocol

Patients underwent MRA with ferumoxytol administered in an appropriate setting under supervision of trained medical personnel and were observed for a minimum of 30 min following termination of ferumoxytol infusion. All studies were performed on a 3.0-T Prisma MRI scanner (Magnetom, Siemens Healthineers) with local phased-array imaging coils in the supine position using standardised protocol similar to that of standard MRA studies with GBCAs.

### Ferumoxytol administration

Ferumoxytol was infused intravenously through a 22-gauge intravenous cannula placed in the antecubital fossa of either arm. Optimal dose was selected on the basis of the minimum effective dose (MED) to achieve diagnostic imaging rather than maximum signal intensity criteria similarly to the ‘As Low As Reasonably Achievable (ALARA) principle’ employed to minimise radiation exposure. A total dose of 4 mg/kg of ferumoxytol (Feraheme; AMAG Pharmaceuticals, Inc.) was delivered up to a maximum of 400 mg. The dosage of ferumoxytol is expressed in terms of milligram of elemental iron, with each millilitre of ferumoxytol containing 30 mg of elemental iron.

In all cases, ferumoxytol was diluted to a concentration no greater than one part ferumoxytol to four parts 0.9% sodium chloride and was administered in 4–7 (median 4) divided controlled infusions (aliquots) until the maximum dose was delivered. Ferumoxytol infusions were delivered by an MRI-compatible infusion pump for precise control over infusion rates which were set at 1 ml/s of diluted ferumoxytol (equal to 6 mg/s of elemental iron) followed by 20 ml of 0.9% sodium chloride at a rate of 1 ml/s. The infusion time for each aliquot ranged up to 18 s depending on body weight and volume of the infusion. These aliquots were delivered with a minimum interval of 5 min between them to allow time for planning and different imaging components to be performed; thus, the total dose was delivered over a minimum of 20 min.

Patients were instructed to immediately alert the operator should they have any discomfort at any time, and were continuously monitored by pulse oximeter (measuring both heart rate and oxygen saturation) while in the MRI scanner and had blood pressure measured before and after infusions. Average scan duration was 30 min.

### Image acquisition

A full range of angiographic sequences were performed depending on the clinical indication; however, for the purposes of this study, the relevant one was a T1-weighted fast low-angle shot (FLASH) sequence of aortoiliac vasculature. This sequence was performed before giving contrast and 60 s after each aliquot of the diluted ferumoxytol was administered without changing the imaging field-of-view between sequences.

### Data analysis

Images were analysed using OsiriX MD (Pixmeo). For quantitative analysis, signal intensities within the vessel lumen were measured using the regions of interest (ROI) placed in the abdominal aorta (AA), inferior vena cava (IVC), psoas muscle, intra-abdominal fat, liver and spleen that were cloned (in terms of position and size) across the acquisitions. An ROI was placed in air outside of the imaged body for noise calculation. The target ROI size was a minimum of 1 cm^2^. If this size could not be achieved in small vessels, the largest practical ROI area for that vessel was assessed. Mean signal intensity (SI; arbitrary units, U) following each dose increment was recorded from all ROIs except those placed in air, from which the standard deviation (SD) was recorded. Contrast-to-noise (CNR) and signal-to-noise (SNR) ratios were calculated. Correlation analyses between CNR and SNR in the AA and IVC and ferumoxytol dose were performed. Assessment of the CNR and SNR was performed independently by two radiologists (MH and GR) with 5 years and > 20 years of experience, respectively, in cardiovascular MR imaging. Following analysis of all images, discordances were adjudicated by discussion and consensus reached.

Descriptive statistics are expressed as means ± SD or numbers and percentages. Four quadratic regression models were used to plot the relationship between cumulative doses of ferumoxytol and CNR and SNR change within AA and IVC, respectively, and estimate the regression coefficients. The open-source statistics environment R (version 3.4.3; The R Foundation) was used for statistical analysis [14].

## Results

A total of 25 patients had angiographic assessment with FeMRA. Two patients were excluded from analysis due to failure to acquire all repeated FLASH sequences because of claustrophobia necessitating abandonment of the scan. From the 23 remaining patients, 13 (57%) required imaging as part of pre-operative kidney transplant assessment, 7 (30%) had clinical manifestations of renovascular disease and 3 (13%) had PVD. Five (22%) patients were on haemodialysis and 18 (78%) had various degrees of renal failure (6 of them had a kidney transplant). The mean age was 59.8 (SD 12.8) years, 20 (87%) were men and almost half of them had diabetic nephropathy. An average of 325 mg (SD 65) of ferumoxytol was administered (Table 1).

**Table 1:** Baseline characteristics

Age (year), mean (SD)	59.8 (12.8)
Female sex, *n* (%)	3 (13.0)
Body weight (kg), mean (SD)	85.4 (22.9)
BMI categories, *n* (%)	
18.5–25	9 (39.1)
25.1–30	4 (17.4)
> 30	10 (43.5)
Cause of ERF	
Diabetes, *n* (%)	11 (47.9)
Renovascular, *n* (%)	3 (13.0)
Other^a^, *n* (%)	6 (26.1)
Unknown, *n* (%)	3 (13.0)
Laboratory values at time of MR imaging	
Haemoglobin (g/L), mean (SD)	106.4 (21.2)
Creatinine (μmoL/L), mean (SD)^b^	359 (131)
eGFR (mL/min/1.73m^2^), mean (SD)^b^	17.2 (7.5)
CKD stage, *n* (%)	
HD	5 (21.7)
5	10 (43.5)
4	8 (34.8)
Dose of ferumoxytol given (mg), mean (SD)	325 (65)

^a^Glomerulonephritis (*n* = 4), reflux nephropathy (*n* = 1), obstructive uropathy (*n* = 1)

^b^Excludes patients on dialysis

*BMI*, body mass index; *ERF*, established renal failure; *eGFR*, estimated glomerular filtration rate; *CKD*, chronic kidney disease; *HD*, haemodialysis

The imaging parameters for the post-contrast breath-hold MRA acquisitions are listed in Table 2. Minor changes to these values were made on an individual basis due to differences in patient body habitus and extracellular fluid status. All subjects completed MRA with ferumoxytol enhancement with no adverse events.

**Table 2:** Pulse sequence parameters for the T1-weighted FLASH sequences

TR (repetition time)	2.88 ms
TE (echo time)	1.04 ms
Flip angle	20°
Number of averages	1
Field of view	400 × 325 mm
Section thickness	1 mm
Voxel dimensions	1.0 × 1.0 × 1.0 mm
Data matrix	243 × 384
Timing of sequence^a^	60 s
Acquisition time	18 s
Mean volume thickness	112
Bandwidth	300 Hz/PX
Parallel imaging acceleration factor	3

^a^After start of contrast infusion

Cumulative doses of 0 (pre-contrast), 1, 2, 3 and 4 mg/kg of ferumoxytol yielded mean CNR of − 41 (± 36), 20 (± 26), 46 (± 29), 90 (± 66) and 86 (± 62) in AA and − 45 (± 37), 4 (± 31), 38 (± 26), 78 (± 46) and 95 (± 71) in IVC, respectively. Mean SNR values were 35 (± 14), 92 (± 63), 97 (± 40), 155 (± 96) and 153 (± 95) in AA and 30 (± 10), 77 (± 43), 89 (± 38), 138 (± 83) and 162 (± 109) in IVC, respectively.

**Fig. 1:**
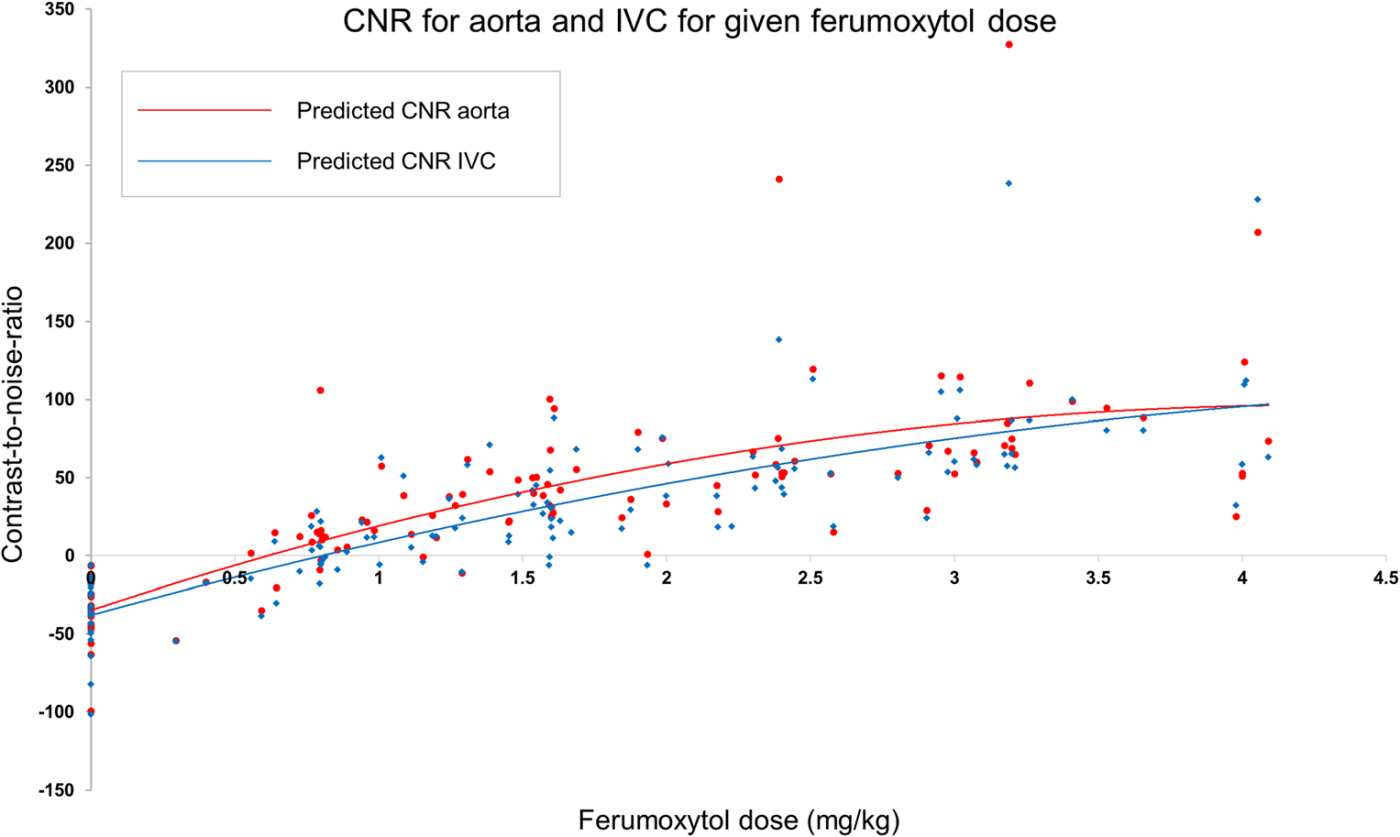
Distribution of CNR measured in abdominal aorta and IVC following incremental doses of ferumoxytol

**Fig. 2:**
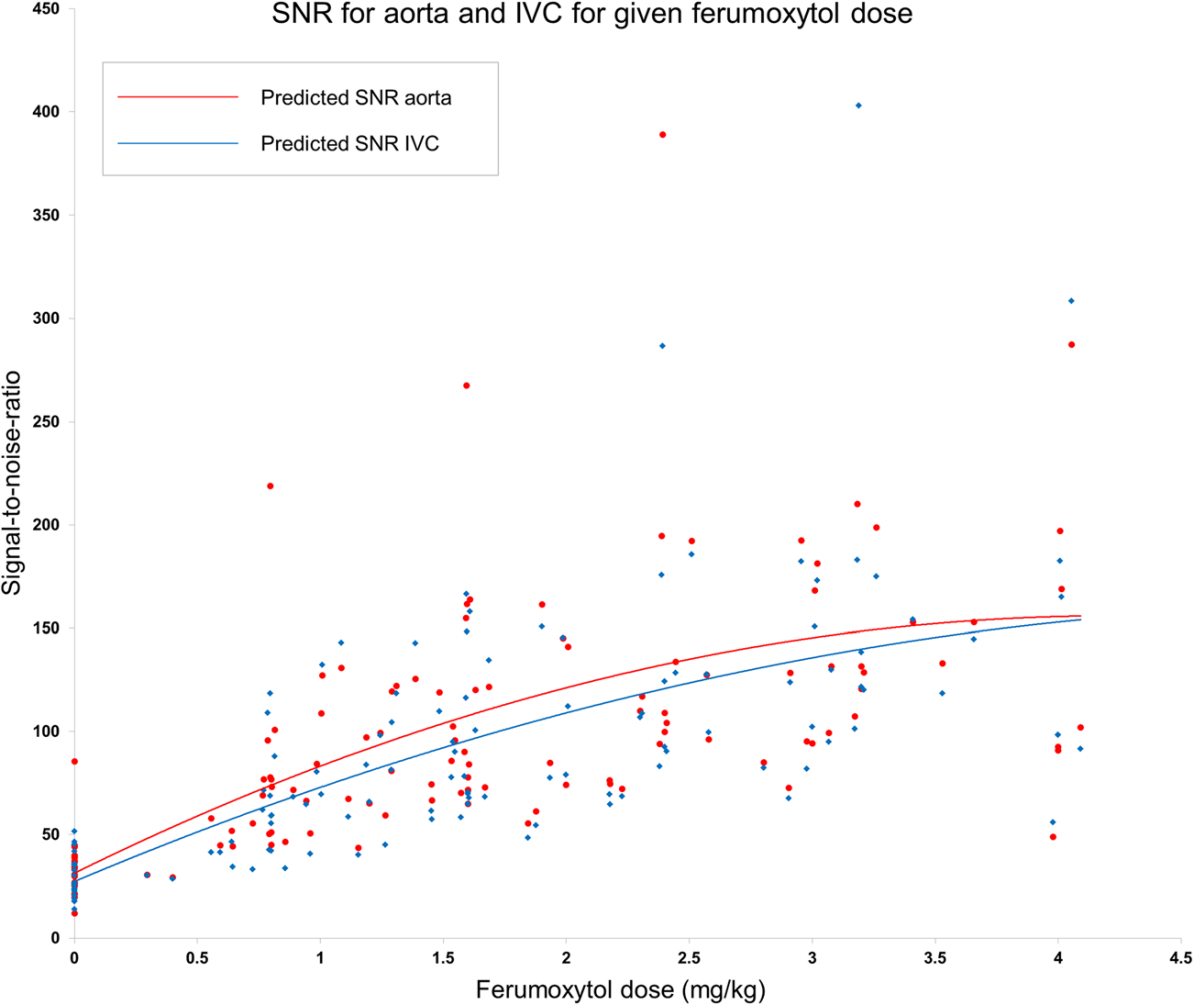
Distribution of SNR measured in abdominal aorta and IVC following incremental doses of ferumoxytol

Figures 1 and 2 demonstrate the distribution of the CNR and SNR, respectively, measured in AA and IVC against the cumulative dose of the administered ferumoxytol. Both graphs demonstrate a parabolic relationship between the administered dose and vascular signal with a line of best fit calculated by quadratic regression analysis.

The calculated relationships for the AA are described below, where *x* represents the ferumoxytol dose and *f*(*x*) the returned signal:

$$ \mathrm{CNR}:f(x)=-6.6{(x)}^2+59.4(x)\kern0.5em -34.1 $$

with an estimated regression co-efficient (*R*^2^) = 0.56 and

$$ \mathrm{SNR}:f(x)=-7.0{(x)}^2+58.9(x)+31.2 $$

with an estimated *R*^2^ = 0.35.

These equations predict peak aortic CNR and SNR at 4 mg/kg ferumoxytol with decrease in signal with higher doses (Tables 3 and 4). However, 85% of peak CNR in AA is predicted to be obtained after administration of 3 mg/kg of ferumoxytol and < 10% of SNR gain is predicted with administration of > 3mg/kg of ferumoxytol (Tables 3 and 4).

**Table 3:** Predicted CNR in abdominal aorta and IVC following ferumoxytol dose increments calculated by the regression equations

Ferumoxytol dose (mg/kg)	Predicted peak CNR in AA (%)	Predicted peak CNR in IVC (%)
0	− 34	− 38
1	19	8
2	58	46
3	85	75
4	98	95
5	98	106
6	85	109

**Table 4:** Predicted SNR in abdominal aorta and IVC following ferumoxytol dose increments calculated by the regression equations

Ferumoxytol dose (mg/kg)	Predicted peak SNR in AA (%)	Predicted peak SNR in IVC (%)
0	31	27
1	83	73
2	121	109
3	145	135
4	155	152
5	151	159
6	133	157

The calculated relationships for the IVC are described below, where *y* represents the ferumoxytol dose and *f*(*y*) the returned signal:

$$ \mathrm{CNR}:f(y)=-4.4{(y)}^2+50.9(y)\kern0.5em -38.1 $$

with an estimated *R*^2^ = 0.65 and

$$ \mathrm{SNR}:f(y)=-4.8{(y)}^2+50.4y+27.2 $$

with an estimated *R*^2^ = 0.44.

**Fig. 3:**
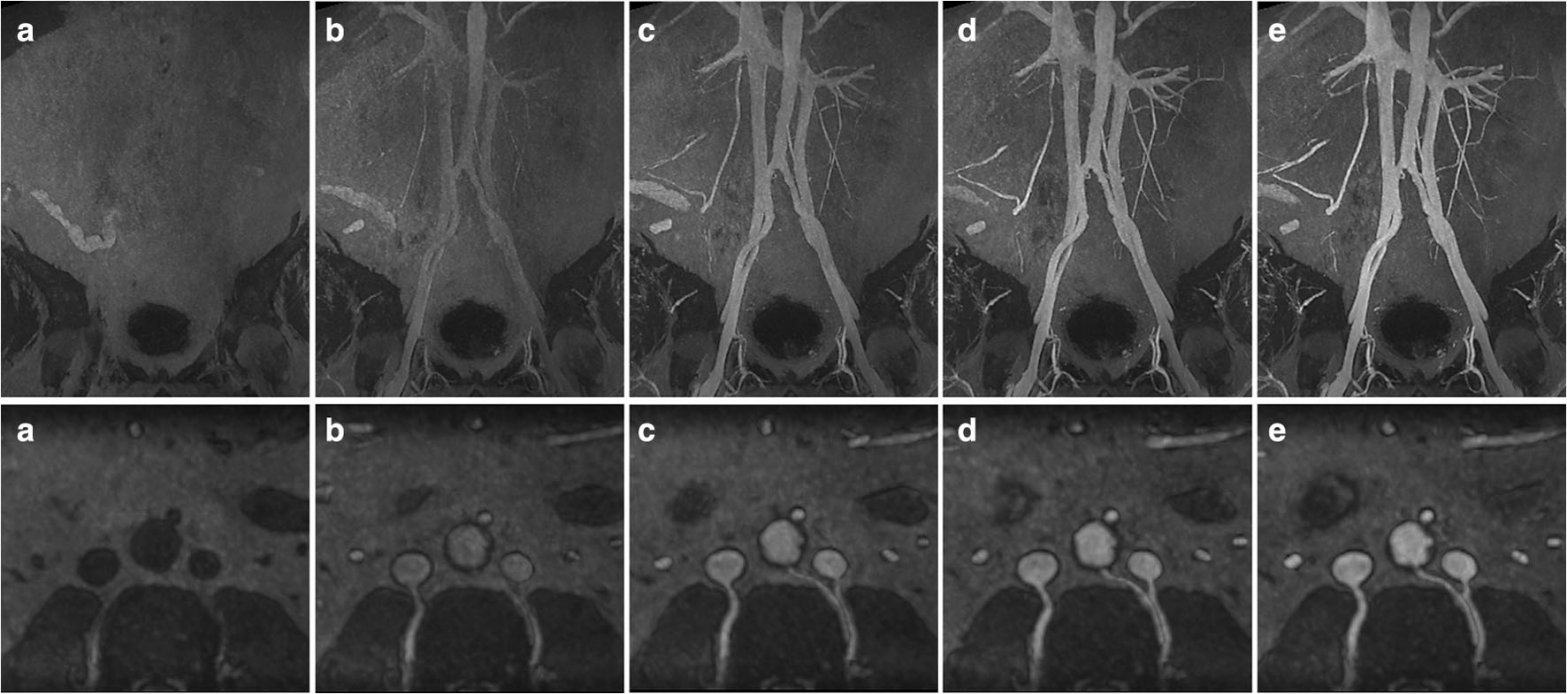
Serial arterial phase maximum intensity projection (MIP) coronal and axial images of abdominal and aortoiliac vasculature after each increment of ferumoxytol. (**a**) Pre-contrast and after administration of (**b**) 1 mg/kg, (**c**) 2 mg/kg, (**d**) 3 mg/kg and (**e**) 4 mg/kg of ferumoxytol

**Fig. 4:**
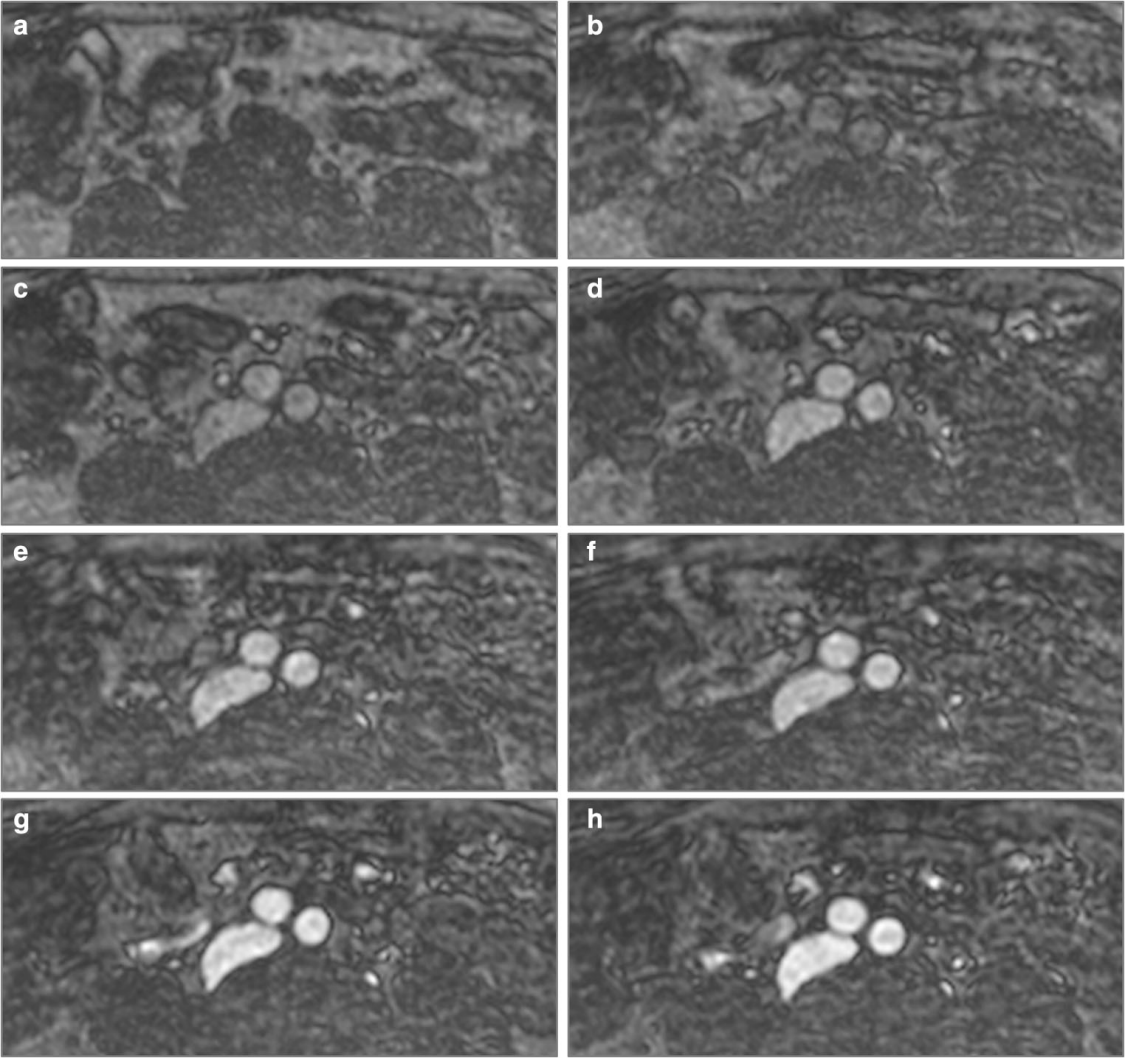
Plane cross-sections of aortic bifurcation before ferumoxytol administration and after serial dose increments. (**a**) Pre-contrast, (**b**) 0.4 mg/kg, (**c**) 0.8 mg/kg, (**d**) 1.2 mg/kg, (**e**) 1.6 mg/kg, (**f**) 2 mg/kg, (**g**) 3 mg/kg and (**h**) 4 mg/kg

**Fig. 5:**
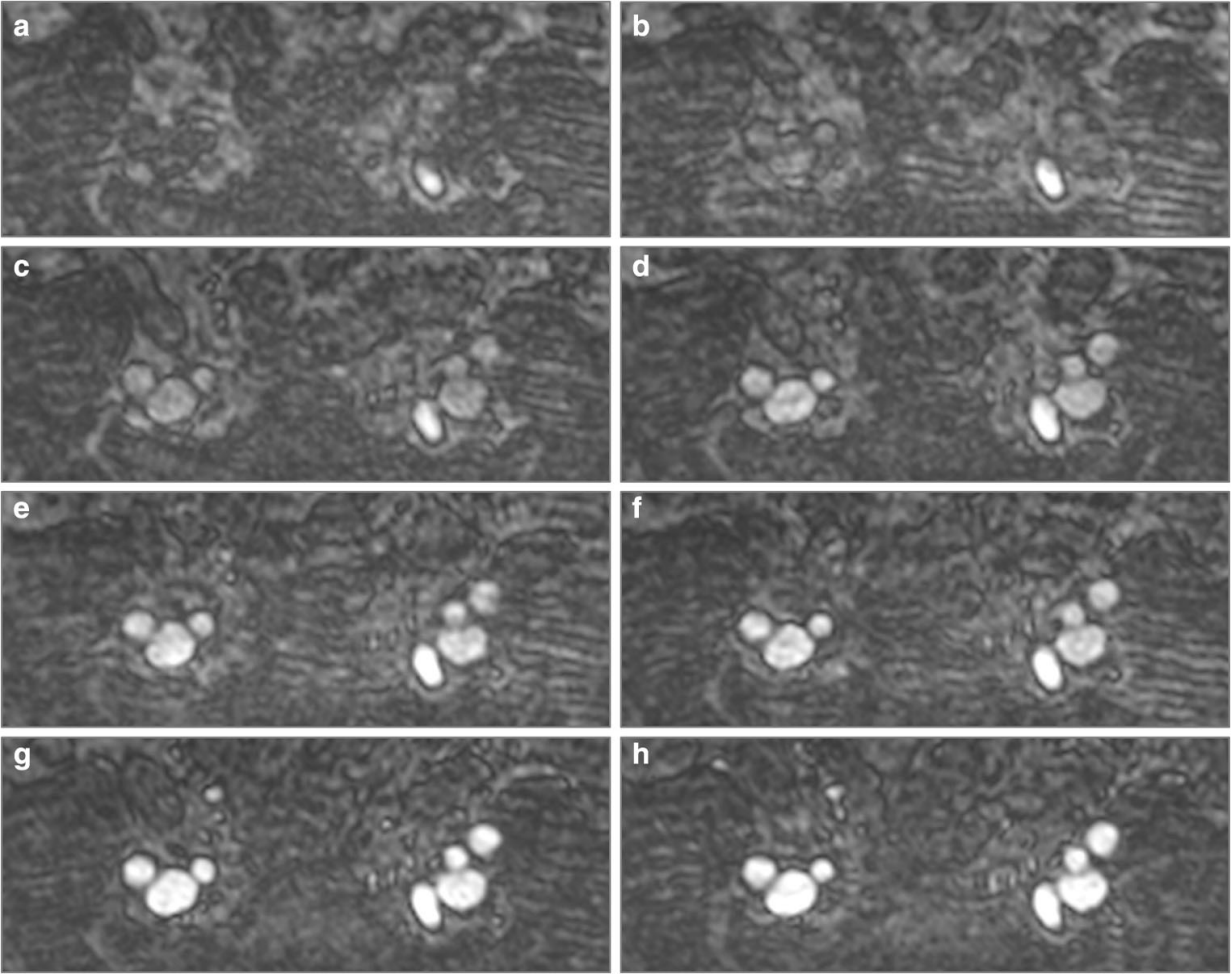
Plane cross-sections below the level of the bifurcation of the common iliac arteries before ferumoxytol administration and after serial dose increments. (**a**) Pre-contrast, (**b**) 0.4 mg/kg, (**c**) 0.8 mg/kg, (**d**) 1.2 mg/kg, (**e**) 1.6 mg/kg, (**f**) 2 mg/kg, (**g**) 3 mg/kg and (**h**) 4 mg/kg

These equations predict peak IVC CNR and SNR at 6 and 5 mg/kg ferumoxytol, respectively, with decrease in signal with higher doses. Still 75% of peak CNR in IVC is predicted to be obtained after administration of 3 mg/kg of ferumoxytol and < 15% of SNR gain is predicted with administration of > 3 mg/kg of ferumoxytol (Tables 3 and 4). Examples of the images obtained are shown in Figs. 3, 4 and 5 where it is obvious that image quality was significantly improved in both arterial and venous compartments following ferumoxytol dose increments up to 3 mg/kg but no visual difference was apparent with higher doses.

**Fig. 6:**
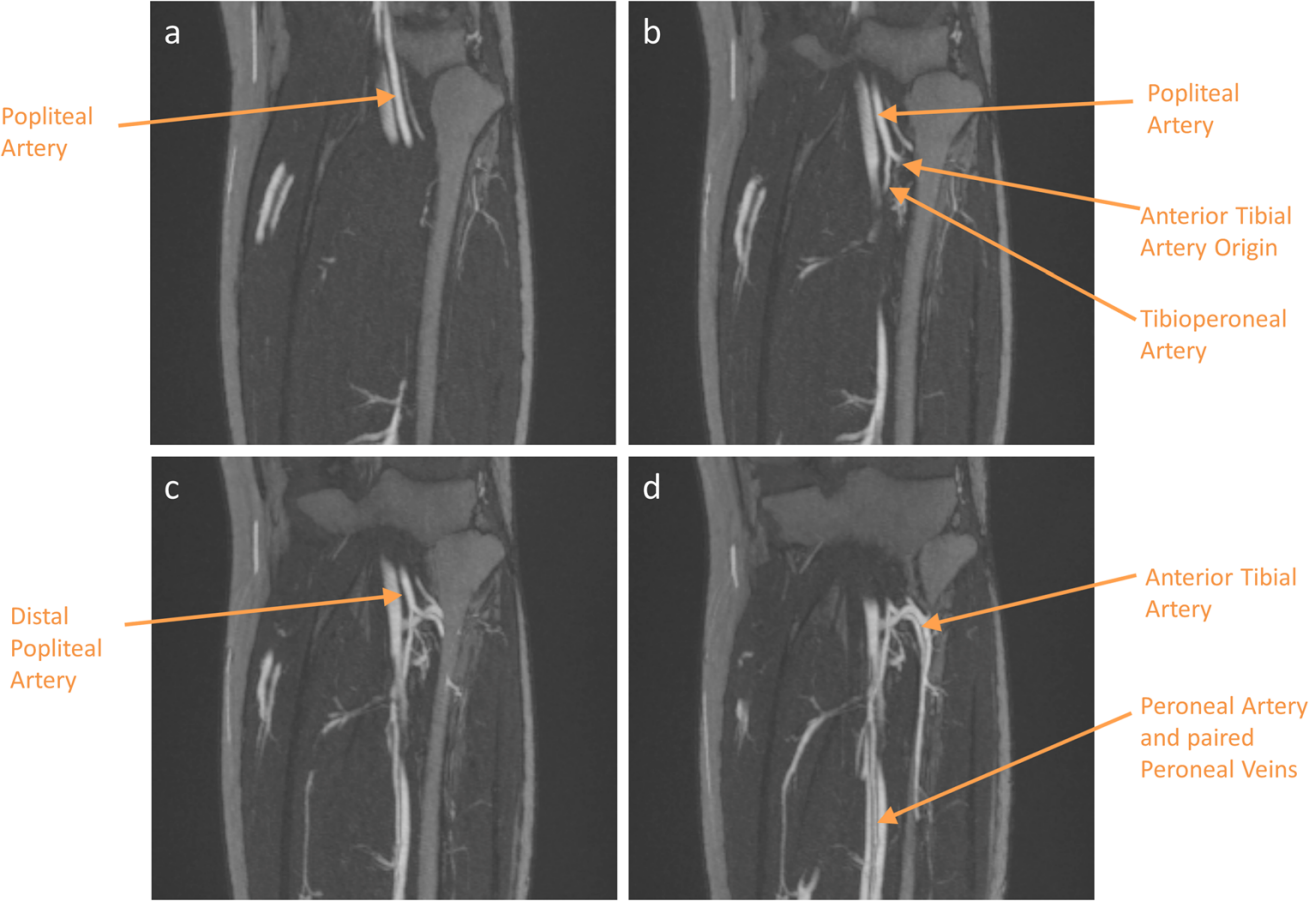
Steady-state coronal thin slab (6mm) maximum intensity projection (MIP) images of the left below knee vasculature at level of tibial plateau and proximal fibula with ferumoxytol-enhanced MRA. Magnified images showing details of distal popliteal and proximal tibial artery branching

With ferumoxytol, arteries and veins can be selectively depicted in a single exam. However, due to the prolonged residence of contrast in the intravascular space, there may be overlay of the arteries and veins, and this is more pronounced in steady-state thick maximum intensity projection (MIP) images of the peripheral vasculature (Fig. 6, Supplemental Video 1).

## Discussion

Ferumoxytol as a contrast agent results in profound shortening of the T1 relaxation time of blood which increases the signal in blood vessels in T1-weighted sequences. However, high concentrations of the agent can cause artefacts [15] because of signal loss through T2* shortening. We used incremental doses of ferumoxytol as an MRI contrast agent for assessing arterial and venous vasculature in patients with CKD to establish the optimal (minimum effective) dose and observed a parabolic relationship between the administered dose and vascular signal. After exceeding a threshold, a negative correlation between the injected dose and the signal intensity was confirmed which is explained by T2* shortening effects at higher doses of ferumoxytol.

Our findings demonstrate dose-dependent improvement in signal with ferumoxytol doses up to 3 mg/kg with no significant benefit (or even loss in signal) with higher doses of contrast. We defined the optimal dose as the minimum effective dose to maximise diagnostic yield while minimising potential for reactions. Although it could be argued that ferumoxytol doses exceeding 3 mg/kg were predicted to achieve better CNR and SNR in the abdominal aorta and inferior vena cava, the gain in signal was minimal, especially considering that a 33% increase in dose above 3 mg/kg is required to yield just 10% increase in signal. Our images were judged as diagnostic when assessed by two readers. It has been argued that peak SNR correlates poorly with the subjective quality rating because it is essentially a pixel-based fidelity measurement method that does not match to the human perception [16]. Hence, the ‘more is better’ approach is not always applicable for SNR. We advocate that 3 mg/kg is an acceptable dose with advantages in terms of protocol simplification, dose saving and minimisation of side effects without compromising diagnostic accuracy.

There are no published dose-finding data on ferumoxytol for vascular imaging with most centres using between 3 and 4 mg/kg based on limited clinical experience. Two studies were identified to formally evaluate the ferumoxytol dose when used as MRI contrast agent, one for MRA and the other for lymphography and lymph node imaging. In a small pilot study including 5 patients who had 3D MRA of the tibial artery trifurcation at 1.5 T, escalating doses of 0.4 mg/kg of ferumoxytol (for a total of 4 mg/kg) were administered. The mean SNR in the arteries increased with higher doses of ferumoxytol; however, a cut-off dose for optimal imaging was not determined [17]. In the non-vascular study investigating ferumoxytol for MR lymphography in 15 patients undergoing prostatectomy with regional lymph node dissection, a dose of 7.5 mg/kg was found to be safe and effective in differentiating between malignant and benign lymph nodes 24 h after ferumoxytol injection [18].

In our initial experience, ferumoxytol has potential clinical utility as a contrast agent for MRI. We have previously reported a case series of kidney transplant candidates to examine the feasibility of acquiring adequate aortoiliac enhancement with FeMRA [11]. Similar MRI and ferumoxytol dosing protocols were used and there is patient overlap between the studies. However, the objective of this current study is fundamentally different and the data analysis used to estimate the optimal dose is novel. Meanwhile, we are testing ferumoxytol in prospective comparative studies of FeMRA versus CT angiography and Doppler ultrasound to assess vasculopathy in CKD patients using similar MR angiography protocols (ClinicalTrials.gov registration number: NCT02997046). As ferumoxytol’s pharmacokinetics are independent of renal function, the proposed dose of 3 mg/kg can be applied universally for MRI irrespectively of renal function. This creates the basis for adoption of this dose in current clinical practice with the caveat that further work is required to translate our protocol to 1.5 T. When given for treatment of anaemia, the licenced dose of ferumoxytol is an initial 510-mg dose followed by a second 510-mg dose 3–8 days later. This corresponds to a dose of approximately 7.3 mg Fe/kg for a 70-kg man or a dose of 14.6 mg Fe/kg over 3–8 days. The 3-mg/kg dose we propose for imaging is substantially lower than this therapeutic dose (approximately a fifth of the full dose for a 70-kg adult) and this should reduce the risk of hypersensitivity reactions while also minimising iron overload if repeated imaging is needed.

Traditional contrast-enhanced MRA employs timed, first-pass imaging of a GBCA bolus, focused on the arterial or venous territory of interest. For the majority of GBCAs, the volume of distribution is the extracellular fluid space which is quickly accessed after a relatively short time within the blood pool; hence, the time window for a vascular phase is very short. Ferumoxytol has a prolonged intravascular half-life of approximately 14.5 h [19] which allows for a longer time window for data acquisition, higher spatial resolution during the equilibrium phase and repeat imaging, if necessary, with negligible loss of intravascular SI [20–22]. FeMRA has been used for both arteriography [5, 7] and venography [8, 10] in patients with CKD and has been shown to provide excellent visualisation of both central and peripheral vessels without the confounding effect of dense vascular calcification on luminal assessment often seen at CT. On the downside, the agent can be present in the blood pool for weeks following administration and months in the reticuloendothelial system, potentially complicating the appearance of follow-up studies [23]. This should be ameliorated with minimised dose.

Ferumoxytol has an iron oxide core encapsulated by a semisynthetic carbohydrate that is substantially different from prior dextran compounds, which diminishes immunogenicity, retards phagocytosis and slows the release of elemental iron from the core. Ferumoxytol has an established safety profile and has no known long-term toxicity [24]. Rates of adverse events have been similar to those seen with iodine-based contrast [24] and the more commonly used intravenous iron formulations [25]. The main concern has been reported cases of anaphylactic-type reactions with rapid therapeutic bolus injections of undiluted compound leading to the recent recommendations by the US FDA for controlled infusion of dilute ferumoxytol [26]. In our study, no adverse events occurred and there was no change in the recorded vital signs during or after administration of ferumoxytol.

This study has notable strengths. We used a consistent protocol with standardised infusion rates and imaging parameters; hence, differences in exposure to ferumoxytol were minimised. Instead of allocating patients into fixed-dose groups, all patients received multiple dose increments allowing for intra- and interpatient dose comparisons. To balance between risk and benefit, the optimal ferumoxytol dose was selected on the basis of maximising signal while maintaining a low-risk threshold rather on the basis of achieving the peak predicted signal.

Our study has several limitations. This is a single-centre study with a relatively small number of patients. However, we used a sequential design to eliminate variations in demographic and clinical factors and detect changes in signal after studying a small set of patients. Translation of the current protocol to 1.5 T needs to be studied but this should be feasible with alterations to echo time etc. to ensure reliable vascular enhancement. Lastly, we have examined dose based upon body weight; however, theoretically, the dose should be based upon the patient’s intravascular blood volume which does not linearly increase with body weight; hence, a relatively lower dose may be appropriate for larger patients.

Using a consistent dosing regimen for contrast administration, we have shown that 3 mg/kg of ferumoxytol is effective for MR angiography in CKD patients in whom there are concerns in regard to standard contrast-based vascular imaging methods. In the era of growing use of ferumoxytol for diagnostic MR applications, this study fills unmet clinical needs by offering an effective dosing regimen.

## Electronic supplementary material

Supplemental Video 1 (MOV 4433 kb)

## Abbreviations

AA: Abdominal aorta
ALARA: As low as reasonably achievable
CIN: Contrast-induced nephropathy
CKD: Chronic kidney disease
eGFR: Estimated glomerular filtration rate
ERF: Established renal failure
FeMRA: Ferumoxytol-enhanced magnetic resonance angiography
IDA: Iron deficiency anaemia
IVC: Inferior vena cava
MED: Minimum effective dose
NSF: Nephrogenic systemic fibrosis
PVD: Peripheral vascular disease
